# Construct Validity of the MOBAK-KG Test for the Assessment of Basic Motor Competencies in Colombian Preschoolers

**DOI:** 10.3390/bs16010146

**Published:** 2026-01-20

**Authors:** Herley Linares-Guzman, Yisel Estrada-Bonilla, Nicolas Martinez-Lopez, Jaime Carcamo-Oyarzun

**Affiliations:** 1 Antonio Jose de Sucre School, Universidad Manuela Beltran, Bogota 110231, Colombia; herleylinares.g@academia.umb.edu.co; 2 Faculty of Health, Universidad Manuela Beltran, Bogota 110231, Colombia; yisel.estrada@docentes.umb.edu.co; 3 CIAM Physical Literacy Research Centre, Universidad de La Frontera, Temuco 4780000, Chile; nicolasesteban.martinez@ufrontera.cl; 4 Department of Physical Education, Sports and Recreation, Universidad de La Frontera, Temuco 4780000, Chile

**Keywords:** motor competencies, MOBAK-KG, initial education, assessment, confirmatory factor analysis

## Abstract

The development of motor competence (MC) is a key objective in preschool education. It is essential to assess MC from a pedagogical perspective using valid and applicable instruments in educational settings. This study aimed to validate the MOBAK-KG test in Colombian preschool children and to describe their motor performance. The sample consisted of 495 children from public schools in Bogotá, Colombia (48.1% girls; M = 5.8 years, SD = 0.60). Factorial validity of the MOBAK-KG test and its correlations with sex, age, and body mass index (BMI) were examined. Confirmatory factor analysis of eight MOBAK-KG items supported a bifactorial structure with object control and self-movement as dimensions (χ^2^ = 33.55; df = 19; *p* = 0.021; CFI = 0.959; RMSEA = 0.039). Including the covariates yielded significant associations in basic motor competencies (χ^2^ = 67.61; df = 33; *p* = 0.0004; CFI = 0.941; RMSEA = 0.046). Results showed sex differences (boys performed better in object control), BMI (negatively related to self-movement), and age (older children performed better). This study demonstrates that the MOBAK-KG test provides a feasible, educationally oriented assessment tool for preschool settings in Colombia. Moreover, it underscores the importance of considering factors such as sex, BMI, and age in the development of motor skills among children.

## 1. Introduction

Early childhood is extremely crucial for developing motor competency (MC) (Berk, 2022). MC is a fundamental component that can promote health through physical activity throughout life, ensuring that children are empowered to actively participate in sport and exercise culture (Herrmann et al., 2019a). Therefore, MC is a relevant educational dimension in general pedagogy (Klafki, 2005) since its influence in the educational field is linked with physical development along with cognitive (Ludyga & Herrmann, 2025; Ludyga et al., 2020; Van der Fels et al., 2015), psychological (Rose et al., 2015) and social (Schierz & Thiele, 2013) development. Therefore, it should be promoted as part of a holistic view of the integral development of school children (Estevan & Barnett, 2018; Leonard, 2016).

MC is defined as a person’s ability to master a set of locomotor, manipulative and stability skills to perform everyday tasks (Utesch & Bardid, 2019). Therefore, it should be understood as a latent functional performance, whose components can be learned and retained in the long term, and which develops based on specific motor demands (Gerlach et al., 2017). MC, as a latent functional performance, is not observed directly. However, it is reflected in the successful outcome of the accomplishment of a given motor task (Carcamo-Oyarzun & Herrmann, 2020; Gerlach et al., 2017). These visible motor activities relate to fundamental motor skills (Gerlach et al., 2017; Haywood & Getchell, 2024). Therefore, these skills cannot be consolidated in later stages when adequate stimulation is absent in childhood (Stodden et al., 2008).

Early education in Colombia constitutes a fundamental right for children under six, promoting holistic development that includes physical competencies, and the national curricula emphasize physical education as essential through tasks focused on movement, and sensory experiences (Ley 1804, 2016; Ministerio de Educación Nacional, 2017, 2019). Thus, it is necessary to recognise that children between the ages of four and six are in the period of developing fundamental motor patterns, where the basic dispositions are acquired to build a sufficiently varied motor repertoire that facilitates the subsequent acquisition of adaptive and competent actions. These can then be flexibly adjusted to different and specific movement contexts (Clark & Metcalfe, 2002) and can be applied to diverse tasks and environmental contexts.

Several factors influence the development of MC, such as sex, age and body mass index (BMI), which are considered as determinants in this study (Barnett et al., 2016; Iivonen & Sääkslahti, 2014; Quintriqueo-Torres et al., 2022). Analyses of motor performances as a function of sex have shown differences in activities related to object control, where boys show higher levels of motor competence than girls (Brian et al., 2019; Robinson et al., 2017). However, meta-analyses have confirmed this association for object control skills, although only with a small effect size (Barnett et al., 2016). On the other hand, the role of sex in activities involving body control has not been clearly established, as some reviews have reported an indeterminate association (Barnett et al., 2016), whereas others have found that girls outperform boys (Iivonen & Sääkslahti, 2014).

Regarding age, motor competence tends to increase as children grow older, and meta-analyses have reported small to medium effect sizes for the association between age and motor competence (Barnett et al., 2016). However, this pattern reflects not only biological maturation but also children’s experiences and interactions with their surroundings (Haywood & Getchell, 2024). With regard to BMI status, systematic reviews have reported strong inverse associations with motor competence (Barnett et al., 2016; Iivonen & Sääkslahti, 2014). However, weight status appears to show indeterminate associations with specific aspects of gross motor competence (Barnett et al., 2016), which warrants further investigation. Likewise, these negative relationships between weight status and motor skills seem to be only emerging in preschool-aged children (Logan et al., 2011; Nervik et al., 2011; Saraiva et al., 2013) and consolidated in primary school students (D’Hondt et al., 2011; V. P. Lopes et al., 2012) where overweight children face more challenges in self-movement motor tasks than normal-weight children. Thus, for example, the study by Quintriqueo-Torres et al. (2022) demonstrates the need to account for biological and sociocultural traits in MC development, emphasizing existing variations among the same group of students in object control tasks, where boys scored higher than girls, with sex being a correlate in this study.

In preschool populations, studies have shown differences according to sex, where boys obtained better values in object control (Herrmann et al., 2019a; Legarra-Gorgoñon et al., 2023) and girls obtained better values in self-movement (Herrmann et al., 2019a). However, sex was not statistically significant in the results of other studies (Legarra-Gorgoñon et al., 2023). The inconsistency in previous findings may be related to differences in measurement tools, cultural context, or sample characteristics. Therefore, it is necessary to examine the cultural sensitivity of assessment protocols and instruments, while also considering the influence of diverse environmental and cultural contexts (L. Lopes et al., 2021). Likewise, there is evidence that MC differences between boys and girls increase with age (Utesch et al., 2018). This highlights the role of age as a correlate of MC, with older children obtaining much higher values in object control and self-movement than younger children (Herrmann et al., 2019a). Additionally, weight status significantly influences motor task performance, negatively affecting motor competence in overweight and obese students (Cumilef-Bustamante et al., 2024). Different investigations have found a weak to moderate negative correlation between MC and BMI (Graf et al., 2004; Logan et al., 2011; V. P. Lopes et al., 2012; Spessato et al., 2013; Wrotniak et al., 2006). This is further evidenced by other studies where children with higher BMI present lower levels of MC (Cliff et al., 2012; Okely et al., 2004).

Hence, the assessment of MC is a primordial element for its development. Although Colombia’s curriculum places strong emphasis on movement and sensory experiences in early childhood, no validated, educationally oriented motor competence instrument has been available for Colombian preschoolers. Therefore, there is a need to evaluate it from an educational perspective (Carcamo-Oyarzun et al., 2022; Carcamo-Oyarzun & Herrmann, 2020), not only to establish an initial diagnosis but also to monitor the learning achieved by the students as a result of the pedagogical interventions (Scheuer et al., 2019). Consequently, at the preschool level, evaluation instruments that are valid and practically applicable in the classroom are required. This should allow the purpose of establishing standards, verification and empirical documentation of the pedagogical effects achieved in this population (Herrmann et al., 2019a).

Among the most widely used instruments for assessing motor skills are the MABC-2 (Henderson et al., 1992) and the KTK (Kiphard & Shilling, 2007). In recent years, the MOBAK test (Herrmann et al., 2019a) has gained popularity due to its ease of administration and its focus on the educational field.

In this context, the objectives of the present study were to (a) establish the construct validity of the MOBAK-KG test in the preschool population of Colombia and (b) describe the motor performance of this age group.

## 2. Materials and Methods

### 2.1. Participants

Four hundred and ninety-five preschoolers aged 4 to 6 years (48.1% girls; Age: M = 5.8, SD = 0.60), enrolled in 25 classes of public educational institutions in the city of Bogota, Colombia, whose population has a medium socioeconomic status, were evaluated using convenience sampling. With respect to potential age effects, two age groups were described separately: a younger subsample aged 49–65 months and an older subsample aged 66–83 months. This split relates to the Colombian preschool system, which includes three grades, and these two are the last ones. Given the convenience sampling from public schools in Bogotá, the findings should not be generalized to all Colombian preschoolers.

The inclusion criteria were enrolment in a public school, age between 4 and 6 years, absence of health restrictions that would prevent them from participating in physical education classes and informed consent provided by their parents, guardians, protectors or caregivers. The study protocol was evaluated and approved by the Research Ethics Committee of the Universidad Manuela Beltrán according to the Act of Approval DCTAFD 24-04 (7 May 2024). The study was conducted in compliance with the Declaration of Helsinki (Asociación Médica Mundial, 2017).

### 2.2. Instruments

#### 2.2.1. MOBAK—KG Test

MOBAK—KG (in German, Motorische Basiskompetenzen im Kindergarten), developed by Herrmann et al. (2019a), was used to assess the status and development of basic motor competencies in early childhood among children aged 4 to 6 years. The MOBAK-KG test comprises eight motor tasks that assess motor competence factors of object control and self-movement. The object control factor is examined using the motor tasks of throwing, catching, bouncing a ball with the hands and dribbling a ball with the foot. The self-movement factor is examined using the motor tasks of balancing, rolling, jumping and running (Herrmann et al., 2020). For each item, students have two attempts, with the exception of the throwing and catching items where they have six attempts. Scoring for these tests is done using a dichotomous scale (0 = failed; 1 = passed), where the number of successful attempts is recorded (never passed = 0 point; passed once = 1 point; passed twice = 2 points). Children have six attempts for the throwing and catching tasks, with the number of successes being scored as 0 (0–2 hits), 1 (3–4 hits) and 2 points (5–6 hits). The score for each item can range between zero and two points, so that the maximum score of each factor could be eight points.

#### 2.2.2. Anthropometric Variables

Height and weight were measured to calculate BMI (kg/m^2^) as a covariate of the MC. Weight status was assessed using age and sex parameters based on World Health Organization classifications (World Health Organization, 2007) for children over 5 years (normal weight: BMI in percentile <85; overweight: BMI in percentile ≥85 to <97; obesity: BMI in percentile ≥97). Height was measured using a SECA 213 stadiometer and weight was assessed using a SECA 803 scale. All preschoolers were assessed barefoot and with minimal clothing.

### 2.3. Procedure

The evaluations were conducted in the spaces utilised for the physical dimension classes, where preschool teachers facilitate the development of children’s motor skills. A group of eight trained evaluators from sport sciences program of the University Manuela Beltran conducted the MOBAK-KG test. These evaluators received training and were subsequently assessed for their level of agreement by analysing a series of reference videos showing children performing the MOBAK tests at different performance levels. Their scores were compared with those of expert trainers, and only those who achieved a high level of agreement were certified as trained assessors to collect data in the study. An 85% concordance was achieved regarding the number of performances used for calibration based on the video recordings. Individual stations were set up for each motor task. Each evaluator was responsible for a group of three to five children and took them through each evaluation station. At each station, the evaluator explained and demonstrated the motor task to be performed. Each preschooler made two attempts in motor tasks (with the exception of throwing and catching, where they had six attempts) without any trial attempt. The estimated time for conducting the tests was 45 to 60 min.

### 2.4. Data Analysis

Descriptive analyses were performed through measures of central tendency and dispersion using the SPSS 29 program (IBM Corp, Armonk, NY, USA). The present analysis further sought to compare the differences between two independent groups in relation to variables of interest (sex and age). The standardised skewness and kurtosis coefficients were utilised to examine the assumptions of normality. Since the data did not meet the assumption of normality and the motor competence variables were treated as ordinal, the nonparametric Mann–Whitney U test (sex and age) and the Kruskal–Wallis test (BMI) were used. Post hoc comparisons were carried out with the Bonferroni test (α set at 0.05) to avoid risk of Type I error. Effect sizes were calculated to quantify the magnitude of the observed differences independently of sample size. For the Mann–Whitney U test, the effect size was estimated using the r statistic. For the Kruskal–Wallis test, the effect size was estimated using eta squared (η^2^). Effect sizes were interpreted according to established benchmarks, with r values of 0.10, 0.30, and 0.50 indicating small, moderate, and large effects, respectively, and η^2^ values of 0.01, 0.06, and 0.14 representing small, moderate, and large effects. The purpose of this procedure was to determine whether there were statistically significant differences between the groups under study. On the other hand, the construct validity of the MOBAK-KG test was assessed through confirmatory factor analyses (CFA) using the statistical software Mplus 7.4 (B. Muthén & Muthén, 2017). In line with previous MOBAK validation studies, we focused on testing the theoretically derived two-factor model and its extension with covariates, and no post hoc model modifications were introduced. Two models were developed: Model 1 aimed to validate the two-factor structure. The items of throwing, catching, bouncing with the hand and driving with the foot were assigned to the object control factor, while the items of balancing, rolling, jumping and running were assigned to the body control factor. Model 2 had the same structure as Model 1, with the addition of sex, BMI and age as covariates. In both models, MOBAK-KG test items were treated on an ordinal scale and the WLSMV (Weighted Least Squares Means and Variance Adjusted) method was used (B. O. Muthén, 1997). The fit of the models was evaluated using the RMSEA (Root Mean Square Error of Approximation) and CFI (Comparative Fit Index), where RMSEA values below 0.06 and CFI values above 0.90 were deemed acceptable (Hu & Bentler, 1999).

## 3. Results

### 3.1. Construct Validity of the MOBAK-KG Test

**Figure 1:**
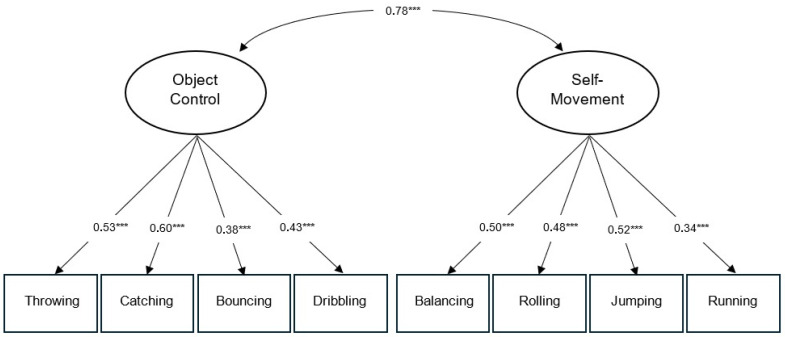
Confirmatory factor analysis with two latent factors (Model 1). Significance level: *** *p* < 0.001, very high significance.

With respect to Model 1, which examines a two-factor structure, the results of the Confirmatory Factor Analysis (CFA) performed for the MOBAK-KG test showed an adequate fit (χ^2^ = 33.55; df = 19; *p* = 0.021; CFI = 0.959; RMSEA = 0.039). The factor loadings fluctuated between 0.34 and 0.60, while the correlation between the two factors was r = 0.78; all these results were statistically significant (see Figure 1 and Table 1).

### 3.2. Relationship Between Sex, Age and BMI Covariates

**Figure 2:**
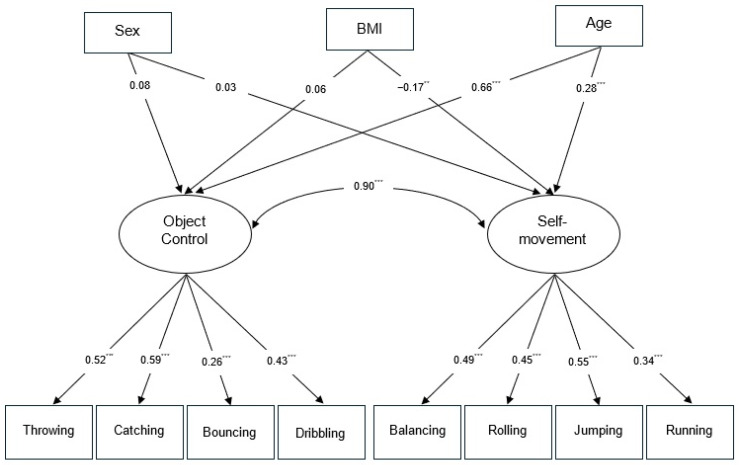
Confirmatory factor analysis with the covariates sex, BMI and age (Model 2). Significance level: ** *p* < 0.01, high significance. *** *p* < 0.001, very high significance.

Regarding Model 2 (Figure 2), which incorporates the covariates of sex, age and BMI, the results of the confirmatory factor analysis indicated adequate fit indices (χ^2^ = 67.61; df = 33; *p* = 0.0004; CFI = 0.941; and RMSEA = 0.046). The covariate sex (binary coding: girls = 1, boys = 2) does not show significant correlations in either of the two factors. The variable age exhibited a positive correlation in both object control and self-movement, indicating that older students obtained higher scores. BMI exhibited a negative correlation only with self-movement (standardized coefficient of −0.17), whereas in object control no statistically significant correlations were found. This suggests that self-movement performance decreased as the BMI increased (Table 2).

Table 3 summaries and compares the global fit indices of the two confirmatory factor analysis models: the two-factor model of object control and body control (Model 1) and the two-factor model including the covariates gender, age, and BMI (Model 2). The table reports the chi-square statistic with its degrees of freedom, the comparative fit index (CFI) as an incremental fit index, and the root mean square error of approximation (RMSEA) as an absolute fit index, indicating adequate overall fit for both models.

### 3.3. Description of the MC of Preschoolers in Colombia

The results regarding performance on the MOBAK-KG test items according to sex have been described below. There was a significant difference (*p* = 0.017) in the total score on the object control factor between girls (M = 3.53, SD = 2.02) and boys (M = 3.97, SD = 2.25). However, when calculating the effect size, it presented a small value (r ≈ 0.10–0.15), indicating that although there is a statistical difference between boys and girls, the practical magnitude of that difference is small (Table 4).

On the other hand, the total score on the self-movement factor was statistically insignificant (*p* = 0.971) between girls (M = 4.16, SD = 2.29) and boys (M = 4.18, SD = 2.20). Moreover, the overall evaluation of the MC, which includes both factors, showed no significant difference according to sex (*p* = 0.145; girls: M = 7.69, SD = 3.57; boys: M = 8.15, SD = 3.80) (Table 4). Both agree with Bonferroni’s correction and the effect size.

Regarding the age covariate, the Mann–Whitney U test was applied to compare the performance in motor skills between the two age groups: children aged 49 to 65 months and children aged 66 to 83 months. The results indicate statistically significant differences in most of the skills evaluated, with a small-to-moderate effect size (r = 0.28). In particular, older children (66 to 83 months) showed better performance in total object control (*p* < 0.001), self-movement (*p* < 0.001) and total MC value (*p* < 0.001) scores (Table 5).

With respect to BMI, Table 6 shows the classification of participants according to WHO BMI categories. No statistically significant differences were found between the groups (*p* = 0.319) in the object control factor. In the case of self-movement, the results indicated a statistically significant difference between the groups (*p* = 0.009). Post hoc comparisons showed that this difference was found specifically between the normal-weight and obese groups (*p* = 0.028), while no significant differences were found between the normal-overweight (*p* = 0.194) and overweight-obese (*p* = 0.652) groups (Table 7).

## 4. Discussion

The present study aimed to establish the construct validity of the MOBAK-KG test in the Colombian preschool population and describe the motor performance of this age group. Considering that the pedagogical evaluation of motor competence in preschool education requires instruments that focus on the functionality of motor performance, the MOBAK-KG test was determined to be an appropriate test for preschool children in Colombia. The present study confirmed the two-factor model (object control and self-movement) presented in the original structure (Herrmann et al., 2019a), verifying its construct validity. The item running showed the lowest factor loading (0.34), a value that can still be considered acceptable, particularly because the item is theoretically important and contributes to the content coverage of the construct. In other MOBAK studies, running has likewise exhibited relatively modest loadings within the self-movement factor, yet it has been retained as a basic indicator of locomotor-related motor competence (Diaz-Alvarado et al., 2025). Likewise, Model 2 demonstrated the relationship of the covariates sex, age and BMI with MC, showcasing results that are consistent with previous studies that have analysed these relationships (Barnett et al., 2016; Carcamo-Oyarzun & Herrmann, 2020; Quintriqueo-Torres et al., 2022; Walter et al., 2024).

When comparing groups on these variables, the present study found sex-related differences, although only with a small effect size, with boys obtaining higher scores in object control, in line with several previous studies (Barnett et al., 2016; Quintriqueo-Torres et al., 2022). On the other hand, some investigations with preschool children have shown that boys and girls do not differ significantly in their motor competence levels. For example, Venetsanou and Kambas (2016), using the Bruininks–Oseretsky Test, reported no sex-based differences in motor performance. These contrasting findings suggest that sex-related patterns in motor competence may depend on contextual factors such as the assessment tool used, opportunities for practice, or cultural expectations. However, these contextual explanations remain hypothetical, as the study did not directly measure practice opportunities or cultural influences. Therefore, our results should be interpreted with caution and considered within the broader variability reported in previous research.

However, there were no significant differences between the scores of boys and girls in self-movement tasks. These results align with previous research (Legarra-Gorgoñon et al., 2023) but contradict other studies that have observed better performance in self-movement skills among girls (Iivonen & Sääkslahti, 2014). This indicates a lack of consensus on the role of sex in locomotion and stability skills. A possible explanation for the lower performance observed among girls in object-control skills may be related to the limited opportunities they often have to engage in ball-related activities; however, this explanation remains hypothetical, as participation in physical or sports activities was not assessed in this study. Previous studies suggest that girls are more frequently encouraged to participate in self-movement activities such as dance (Wu et al., 2025) or gymnastics (Adamo et al., 2016; Temple et al., 2014), which may reduce their exposure to activities that support the development of object-control competencies. However, this interpretation should be considered cautiously, as the current dataset does not include measures of participation opportunities and therefore cannot confirm this mechanism.

With respect to age, older children obtained much higher values in both factors than younger children, although only with a small effect size, coinciding with previous literature (Herrmann et al., 2021; Strotmeyer et al., 2020). Consequently, this interpretation of test performances according to age occurs because younger children have just started preschool, while older children are about to start elementary school. Therefore, they have had more opportunities to experience their skills and accumulate diverse motor experiences, which can allow them to perform better (Herrmann et al., 2019b). Therefore, these differences would be expected and illustrate that the level of motor performance of preschoolers should always be interpreted as a function of age (Kühnis et al., 2019).

Regarding weight status, BMI showed a weak positive relationship in object control and a negative relationship in self-movement in the present study, coinciding with the evidence found in previous studies (Carcamo-Oyarzun et al., 2025a; Carcamo-Oyarzun & Herrmann, 2020; Quintriqueo-Torres et al., 2022). In contrast to object control skills, which tend to be more static, locomotor and stability skills involve moving or controlling a greater body mass, so excess weight hinders functional movement (Duncan et al., 2013). It is important to consider that these negative correlations can be projected to the course of elementary school (Spessato et al., 2013). Therefore, it is essential to take measures to avoid falling into a negative spiral generated by low MC and low levels of participation in physical activities (Stodden et al., 2008).

When contrasting international studies with the results of the present study focused on preschoolers in Colombia, it is possible to establish that the performance of Colombian children is mostly lower than that of other studies that have used the MOBAK-KG test. For comparisons with the Swiss sample, effect sizes were approximated from the reported means and 95% confidence intervals because standard deviations were not available (Herrmann et al., 2019b); therefore, these Cohen’s d values should be interpreted as rough estimates of the magnitude of between-country differences rather than precise effects. Regarding the data obtained in object control, Colombian preschoolers presented lower values (M = 3.76, SD = 2.15) in contrast to the results observed among Swiss preschoolers (M = 4.39, 95% CI = 4.18–4.59; approximate d ≈ −0.30) (Herrmann et al., 2019b), and higher than Spanish preschoolers (M = 2.60, SD = 1.90; d ≈ 0.57) (Legarra-Gorgoñon et al., 2023). In self-movement, Colombian preschoolers (M = 4.17, SD = 2.24) showed lower results than Swiss preschoolers (M = 4.43, 95% CI = 4.21–4.64; approximate d ≈ −0.12) (Herrmann et al., 2021), and superior to Spanish preschoolers (M = 3.90, SD = 2.40; d ≈ 0.12) (Legarra-Gorgoñon et al., 2023). Regarding the results obtained in the total MC, Colombian children presented lower values (M = 7.93) with respect to Swiss children (M = 8.82, 95% CI = 8.45–9.20; approximate d ≈ −0.29) (Herrmann et al., 2019b), and higher than Spanish children (M = 6.40, SD = 3.80; d ≈ 0.49) (Legarra-Gorgoñon et al., 2023), highlighting the need to consider country or culture as a correlate of motor competence.

This study has some limitations. First, the sample of participants, as a convenience sample concentrated in one city in Colombia, is not representative of the population, and we cannot extrapolate the results to all Colombian preschool children. In addition, there were limitations in terms of time, financial resources, and access to the target population. Another methodological limitation is the absence of a formal inter-rater reliability assessment, which we attempted to mitigate through rater training and video-based evaluation to standardize the assessment criteria. Therefore, future studies should include a larger sample from multiple cities. Second, although the cross-sectional design used is appropriate for describing the level of motor competence at the moment of assessment, this type of design does not allow for the examination of developmental trajectories or changes over time. Therefore, longitudinal studies would be valuable for understanding the evolution of motor competence during the early years of schooling and for generating evidence that contributes to establishing normative values specific to the Colombian preschool population. Future longitudinal studies could also support the establishment of Colombian age- and sex-specific normative values for motor competence, further strengthening the applied relevance of this line of research.

The findings of this study support the construct validity of the MOBAK-KG test for assessing motor competence in Colombian preschoolers, as reflected in its adequate model fit, significant factor loadings, and coherent two-factor structure. Overall, the children demonstrated moderate levels of motor competence, with higher scores in self-movement than in object control. Regarding sociodemographic and anthropometric covariates, age emerged as a consistent and meaningful predictor, with older children showing significantly better performance across both factors and the total MC score. In contrast, sex differences were minimal: although boys showed slightly higher object-control scores, these differences did not remain significant after Bonferroni correction and were accompanied by small effect sizes, while no sex differences were observed in self-movement or total MC. Finally, BMI showed no association with object control, but higher BMI was related to lower performance in self-movement, particularly when comparing children with obesity to those with normal weight. Together, these results confirm the utility of the MOBAK-KG for identifying variability in early motor competence and highlight the importance of considering age and BMI, while interpreting sex differences with caution, when evaluating motor development in preschool populations.

Early childhood is crucial for gaining essential experiences with movement and developing basic motor competencies (Sudgen & Soucie, 2017; Meinel & Schnabel, 2007). The results of this study contribute to the literature by validating a relevant test to assess MC among preschoolers in Colombia along with providing a description of their motor performance. The validation of the MOBAK-KG test for Colombian preschoolers has important practical implications for physical education and early childhood physical activity promotion. It provides a standardized, low-cost, and easy-to-use tool for teachers to assess motor competence in 4- to 6-year-olds, enabling early identification of motor strengths and needs and guiding context-specific pedagogical and intervention decisions. Its systematic use allows motor development to be monitored over time and the impact of curricular initiatives or projects to be evaluated, while the availability of a locally validated instrument generates national evidence on motor competence that can inform curriculum guidelines and public policies tailored to the motor realities of Colombian children.

The MOBAK-KG test offers an easy way to assess children’s motor development from an educational viewpoint (Carcamo-Oyarzun et al., 2022; Scheuer et al., 2019) and assess the efficiency of the learning methods (e.g., at the conclusion of a teaching unit or school program) and aid in building motor competencies. Through its application, teachers will be able to design programs to promote movement and physical activity (Saamong et al., 2024) as well as, to identify the motor tasks in which their students present greater difficulty. This will allow them to plan and elaborate didactic strategies that foster necessary learning so that their students can successfully overcome the motor demands presented to them, both in physical education classes and in their daily lives, thereby developing physical literacy (Carcamo-Oyarzun et al., 2025a, 2025b).

## 5. Conclusions

This study provides evidence for the construct validity of the MOBAK-KG test in Colombian preschoolers, confirming its original two-factor structure (object control and self-movement). The results indicate moderate overall competence, with better self-movement than object-control performance, age as a positive correlate, minimal sex differences, and higher BMI selectively associated with poorer self-movement, in line with patterns reported in international MOBAK research while highlighting cross-country variability in performance levels. Together, these findings extend the MOBAK evidence base to the Colombian context and provide a standardized, low-cost, pedagogically oriented tool for diagnosing early motor competence, monitoring development, and evaluating movement- and physical-activity-promotion initiatives in preschool education. Future research should incorporate larger and more diverse samples and use longitudinal designs that integrate contextual variables (e.g., practice opportunities and cultural factors) to refine normative references and clarify developmental pathways linking motor competence, physical activity, and physical literacy in early childhood.

## Figures and Tables

**Table 1:** Item loadings and factor correlations for Model 1.

Factor	Item	Factor Loadings	SE	*p*
Object Control	Throwing	0.53	0.049	<0.001
	Catching	0.60	0.048	<0.001
	Bouncing	0.38	0.052	<0.001
	Dribbling	0.43	0.051	<0.001
Self-movement	Balancing	0.50	0.051	<0.001
	Rolling	0.48	0.052	<0.001
	Jumping	0.52	0.050	<0.001
	Running	0.34	0.054	<0.001
Object Control ↔ Self-movement		**Correlation**	**SE**	***p***
		**0.78**	0.066	<0.001

**Table 2:** Standardized coefficients for the covariate associations in Model 2.

Factor	Covariate	Factor Loadings	SE	*p*
Object Control	Sex	0.08	0.060	0.181
	Age	0.66	0.047	<0.001
	BMI	0.06	0.054	0.282
Self-movement	Sex	0.03	0.074	0.634
	Age	0.28	0.059	<0.001
	BMI	−0.17	0.060	0.004

**Table 3:** Fit indices for the two-factor (model 1) and two-factor with covariables (model 2) CFA models.

Model	χ^2^	*df*	CFI	RMSEA
Model 1 two-factor structure of MOBAK KG	33.55	19	0.959	0.039
Model 2 two-factor structure of MOBAK KG with the covariates of sex, age and BMI	67.61	33	0.941	0.046

**Table 4:** Descriptive data for performance on MOBAK-KG test items according to sex.

Item	Total (n = 495)			Girls (n = 238)			Boys (n = 257)			*p*	r
	Mean	SD	95% CI	Mean	SD	95% CI	Mean	SD	95% CI		
Throwing	0.90	0.74	[0.83; 0.96]	0.79	0.72	[0.69; 0.88]	1.00	0.74	[0.91; 1.10]	0.001 **	0.148
Catching	1.29	0.81	[1.22; 1.36]	1.30	0.80	[1.20; 1.40]	1.28	0.83	[1.18; 1.39]	0.947	−0.003
Bouncing	0.82	0.87	[0.74; 0.90]	0.92 **	0.87	[0.81; 1.03]	0.72	0.87	[0.62; 0.83]	0.009	−0.117
Dribbling	0.75	0.90	[0.67; 0.83]	0.52	0.80	[0.42; 0.62]	0.96	0.94	[0.85; 1.08]	<0.001 ***	0.148
**Total Object control**	**3.76**	**2.15**	**[3.57; 3.95]**	**3.53**	**2.02**	**[3.27; 3.78]**	**3.97**	**2.25**	**[3.70; 4.25]**	**0.017** *	**0.107**
Balancing	0.60	0.84	[0.53; 0.68]	0.63	0.83	[0.52; 0.73]	0.58	0.85	[0.47; 0.68]	0.355	−0.042
Rolling	1.29	0.90	[1.21; 1.37]	1.25	0.90	[1.13; 1.36]	1.33	0.90	[1.22; 1.45]	0.214	0.056
Jumping	1.10	0.90	[1.02; 1.18]	1.27	0.84	[1.16; 1.38]	0.94	0.92	[0.83; 1.06]	<0.001 ***	−0.148
Running	1.18	0.87	[1.10; 1.25]	1.02	0.88	[0.91; 1.13]	1.32	0.84	[1.22; 1.43]	<0.001 ***	0.148
**Total Self-movement**	**4.17**	**2.24**	**[3.97; 4.37]**	**4.16**	**2.29**	**[3.87; 4.46]**	**4.18**	**2.20**	**[3.91; 4.45]**	**0.971**	**0.002**
**Total** **Basic Motor Competencies**	**7.93**	**3.69**	**[7.60; 8.26]**	**7.69**	**3.57**	**[7.23; 8.14]**	**8.15**	**3.80**	**[7.68; 8.62]**	**0.145**	**0.066**

Mann–Whitney U Test *** *p* < 0.001, very high significance; ** *p* < 0.01, high significance; * *p* < 0.05, significant. r values: <0.10 no significant, 0.10 small, 0.30 moderate, and 0.50, large effects.

**Table 5:** Distribution of means, standard deviations and confidence intervals for each of the MOBAK—KG test items, according to age.

Item	49 to 65 Months (n = 155)			66 to 83 Months (n = 340)				
	M	SD	95% CI	M	SD	95% CI	*p*	r
Throwing	0.61	0.62	[0.51; 0.71]	1.03	0.75	[0.95; 1.11]	<0.001 ***	0.26
Catching	0.86	0.84	[0.73; 1.00]	1.49	0.73	[1.41; 1.56]	<0.001 ***	0.35
Bouncing	0.74	0.86	[0.61; 0.88]	0.85	0.88	[0.76; 0.95]	0.191	0.06
Dribbling	0.48	0.76	[0.36; 0.60]	0.87	0.93	[0.77; 0.97]	<0.001 ***	0.19
**Total Object control**	**2.70**	**1.98**	**[2.39; 3.02]**	**4.24**	**2.05**	**[4.02; 4.46]**	<0.001 ***	**0.33**
Balancing	0.53	0.79	[0.40; 0.65]	0.64	0.86	[0.54; 0.73]	0.260	0.05
Rolling	1.15	0.92	[1.00; 1.30]	1.36	0.88	[1.27; 1.45]	0.014 *	0.11
Jumping	0.85	0.88	[0.71; 0.98]	1.21	0.88	[1.12; 1.31]	<0.001 ***	0.19
Running	1.10	0.87	[0.96; 1.23]	1.21	0.87	[1.12; 1.31]	0.140	0.07
**Total Self-movement**	**3.62**	**2.19**	**[3.27; 3.97]**	**4.42**	**2.22**	**[4.19; 4.66]**	<0.001 ***	**0.17**
**Total** **Basic Motor Competencies**	**6.32**	**3.57**	**[5.76; 6.89]**	**8.66**	**3.52**	**[8.29; 9.04]**	<0.001 ***	**0.28**

Mann–Whitney U Test: *** *p* < 0.001, very high significance; * *p* < 0.05, significant. r values: <0.10 no significant, 0.10 small, 0.30 moderate, and 0.50, large effects.

**Table 6:** Distribution of means, standard deviations and confidence intervals for each of the MOBAK—KG test items by sex and WHO BMI categories.

	Item	Normal Weight BMI in Percentile <85			Overweight BMI in Percentile ≥85 to <97			Obesity BMI in Percentile ≥97			Total
		n	Mean	SD	n	Mean	SD	n	Mean	SD	n
Boys	Object Control	175	4.19	2.20	39	4.31	1.89	16	4.94	1.81	230
	Self-movement		4.48	2.18		4.44	2.02		3.06	2.41	
	Total BMC		8.67	3.74		8.74	3.17		8.00	3.58	
Girls	Object Control	180	3.67	1.97	24	3.54	2.15	8	4.00	2.39	212
	Self-movement		4.49	2.19		3.25	2.23		3.63	3.07	
	Total BMC		8.17	3.37		6.79	3.67		7.63	4.37	
Total Object Control		355	3.93	2.10	63	4.02	2.01	24	4.63	2.02	442
Total Self-movement			4.49	2.18		3.98	2.17		3.25	2.59	
Total BMC			8.41	3.56		8.00	3.47		7.88	3.77	

**Table 7:** Distribution of means and standard deviations of the MOBAK—KG test, according to BMI.

	Normal Weight (n = 355)		Overweight (n = 63)		Obesity (n = 24)				
Item	Mean	SD	Mean	SD	Mean	SD	*p*	η^2^	Post Hoc Analysis
**Total** **Object Control**	3.93	2.10	4.02	2.01	4.63	2.02	0.319		-
**Total Self-movement**	4.49	2.18	3.98	2.17	3.25	2.59	0.009 *	0.017	- Normal vs. Obesity: *p* = 0.028 * - Normal vs. overweight: *p* = 0.194 - Overweight vs. Obesity: *p* = 0.652
**Total** **Basic Motor Competencies**	8.41	3.56	8.00	3.47	7.88	3.77	0.397		-

* *p* < 0.05, significant (normal weight-obesity). η^2^ values: 0.01 small, 0.06 moderate, and 0.14 large effects.

## Data Availability

The data presented in this study are available on request from the corresponding author. The data are not publicly available due to privacy and ethical restrictions, as participants are minors.
